# Supplementary material for: ProteinShader: illustrative rendering of macromolecules
Source: BMC Struct Biol. 2009 Mar 30;9:19. doi: 10.1186/1472-6807-9-19 (PMC2672931; doi:10.1186/1472-6807-9-19)
Supplement: Additional file 1 — ProteinShader program without source code. This compressed file contains the complete ProteinShader program including associated libraries, but no source code. A README.txt file gives an overview of the ProteinShader distribution, and the index.html file in the help subdirectory has directions on getting started with the program as well as a set of tutorials. [file 1472-6807-9-19-S1.zip › ProteinShader-beta-0_9_4-binary/help/api/org/proteinshader/structure/io/demos/SegmentDemo.html]

SegmentDemo (ProteinShader API)


|  |  |  |
| --- | --- | --- |
| **Overview** \| **Package** \| **Class** \| **Use** \| **Tree** \| **Deprecated** \| **Index** \| **Help** | |  |
| **PREV CLASS**   NEXT CLASS | **FRAMES**    **NO FRAMES**     **All Classes** |  |
| SUMMARY: NESTED \| FIELD \| CONSTR \| METHOD | DETAIL: FIELD \| CONSTR \| METHOD |  |


---


## org.proteinshader.structure.io.demos Class SegmentDemo

```
java.lang.Object
  org.proteinshader.structure.io.demos.SegmentDemo
```

---

``` public class SegmentDemo extends Object ```

Prints Segment data for troubleshooting.
USAGE:
java -cp . path/SegmentDemo inputFilename outputFilename
where path = edu/harvard/fas/jrweber/molecular/structure/io
After reading 'inputFilename' and creating a Structure, the
Structure will be written as an object dump to a file named
'outputFilename.out'.
Any bad lines found while parsing the Structure will be printed to
standard error. On Unix, output to standard error can be redirected
to a file by placing ' 2> error\_filename' as the last thing on the
command line used to invoke the program.

---

| **Field Summary** | |
| --- | --- |
| `static String` | `PATH` |
| `static String` | `usageMessage` |


**Constructor Summary**

`SegmentDemo()`
  
          Constructs a SegmentDemo.


| **Method Summary** | |
| --- | --- |
| `String` | `getFilenameOrDie(String[] cmdLineArgs, int index)`             Returns the requested command line argument if it is present. |
| `static void` | `main(String[] args)`             Uses a PDBStructureReader to read a PDB file and create a Structure, and then uses a WriterVisitor to write the contents of the Structure to an output file. |
| `void` | `printBadLines(List<String> badLines)`             Prints the bad lines from the PDB file to standard out. |
| `void` | `writeStructure(Structure structure, String filename)`             Writes a Structure to a file by using a SFWriterVisitor. |

**Methods inherited from class java.lang.Object**

`clone, equals, finalize, getClass, hashCode, notify, notifyAll, toString, wait, wait, wait`

**Field Detail**

### PATH

```
public static final String PATH
```

**See Also:**: Constant Field Values

---


### usageMessage

```
public static final String usageMessage
```

**See Also:**: Constant Field Values


**Constructor Detail**

### SegmentDemo

```
public SegmentDemo()
```

:   Constructs a SegmentDemo.


**Method Detail**

### writeStructure

```
public void writeStructure(Structure structure,
                           String filename)
                    throws VisitorException
```

:   Writes a Structure to a file by using a SFWriterVisitor. If
    filename does not exist, it will be created. If it already
    exists, it will be overridden.

    :   **Parameters:**: `structure` - the Structure to write.: `filename` - the name of the file to write to. **Throws:**: `VisitorException` - if an IO error occurs.

---


### printBadLines

```
public void printBadLines(List<String> badLines)
```

:   Prints the bad lines from the PDB file to standard out. To save
    bad lines to a file, use the redirection command for standard out,
    ' > filename'.

    :   **Parameters:**: `badLines` - the bad lines to print.

---


### getFilenameOrDie

```
public String getFilenameOrDie(String[] cmdLineArgs,
                               int index)
```

:   Returns the requested command line argument if it is present.
    Otherwise, prints a usage message to standard out and then
    terminates the program.

    :   **Parameters:**: `cmdLineArgs` - the command line arguments.: `index` - the index position to get the filename from.

---


### main

```
public static void main(String[] args)
```

:   Uses a PDBStructureReader to read a PDB file and create a
    Structure, and then uses a WriterVisitor to write the contents of
    the Structure to an output file.


---


|  |  |  |
| --- | --- | --- |
| **Overview** \| **Package** \| **Class** \| **Use** \| **Tree** \| **Deprecated** \| **Index** \| **Help** | |  |
| **PREV CLASS**   NEXT CLASS | **FRAMES**    **NO FRAMES**     **All Classes** |  |
| SUMMARY: NESTED \| FIELD \| CONSTR \| METHOD | DETAIL: FIELD \| CONSTR \| METHOD |  |


---

# *Copyright © 2007-2008*
